# Cell-free DNA in Human Follicular Microenvironment: New Prognostic Biomarker to Predict *in vitro* Fertilization Outcomes

**DOI:** 10.1371/journal.pone.0136172

**Published:** 2015-08-19

**Authors:** Sabine Traver, Elodie Scalici, Tiffany Mullet, Nicolas Molinari, Claire Vincens, Tal Anahory, Samir Hamamah

**Affiliations:** 1 CHU Montpellier, INSERM U1203, Saint-Eloi Hospital, Institute of Regenerative Medicine and Biotherapy, Montpellier, France; 2 Montpellier 1 University, UFR of Medicine, Montpellier, France; 3 ART-PGD Department, Arnaud de Villeneuve Hospital, CHU Montpellier, Montpellier, France; 4 UMR 1049, DIM, CHRU, Montpellier, France; Utah State University, UNITED STATES

## Abstract

Cell-free DNA (cfDNA) fragments, detected in blood and in other biological fluids, are released from apoptotic and/or necrotic cells. CfDNA is currently used as biomarker for the detection of many diseases such as some cancers and gynecological and obstetrics disorders. In this study, we investigated if cfDNA levels in follicular fluid (FF) samples from *in vitro* fertilization (IVF) patients, could be related to their ovarian reserve status, controlled ovarian stimulation (COS) protocols and IVF outcomes. Therefore, 117 FF samples were collected from women (n = 117) undergoing IVF/Intra-cytoplasmic sperm injection (ICSI) procedure and cfDNA concentration was quantified by ALU-quantitative PCR. We found that cfDNA level was significantly higher in FF samples from patients with ovarian reserve disorders (low functional ovarian reserve or polycystic ovary syndrome) than from patients with normal ovarian reserve (2.7 ± 2.7 ng/μl versus 1.7 ± 2.3 ng/μl, respectively, p = 0.03). Likewise, FF cfDNA levels were significant more elevated in women who received long ovarian stimulation (> 10 days) or high total dose of gonadotropins (≥ 3000 IU/l) than in women who received short stimulation duration (7–10 days) or total dose of gonadotropins < 3000 IU/l (2.4 ± 2.8 ng/μl versus 1.5 ± 1.9 ng/μl, p = 0.008; 2.2 ± 2.3 ng/μl versus 1.5 ± 2.1 ng/μl, p = 0.01, respectively). Finally, FF cfDNA level was an independent and significant predictive factor for pregnancy outcome (adjusted odds ratio = 0.69 [0.5; 0.96], p = 0.03). In multivariate analysis, the Receiving Operator Curve (ROC) analysis showed that the performance of FF cfDNA in predicting clinical pregnancy reached 0.73 [0.66–0.87] with 88% specificity and 60% sensitivity. CfDNA might constitute a promising biomarker of follicular micro-environment quality which could be used to predict IVF prognosis and to enhance female infertility management.

## Introduction

During *in vitro* fertilization (IVF) procedures, the ovarian reserve status must be evaluated to optimize the ovarian response to stimulation [1–3]. Indeed, controlled ovarian stimulation (COS) by gonadotropin treatment should be adjusted based on the patient’s ovarian reserve status [4]. However, the biomarkers currently used to assess the ovarian reserve, such as anti-Müllerian hormone (AMH) and antral follicle count (AFC), are not sufficiently reliable. Sometimes, these two parameters can be inconsistent because of the lack of standardization between practitioners or laboratories [5–9].Therefore, the identification of new biomarkers that reflect more accurately the ovarian reserve status and the expected response to gonadotropin treatments might increase IVF success by improving personalized care.

DNA fragments are the result of apoptotic or necrotic events and can be easily detected in blood and in other body fluids [10, 11], including follicular fluid (FF) [12]. Cell-free DNA (cfDNA) level is increased in some cancers and other severe diseases (for instance, some gynecological and obstetrics disorders) and is already used as a non-invasive biomarker for their early detection and/or prognosis [13–15]. Moreover, we have previously demonstrated that cfDNA level in individual FF samples reflects the proportion of apoptotic and necrotic cells inside ovarian follicles and varies according to the follicular size during COS [12]. For these reasons, FF cfDNA could represent a new biomarker of follicular microenvironment quality, and consequently could be affected by ovarian reserve disorders and by the different COS protocols.

As oocyte quality and its microenvironment affect early embryo development [16], many studies have tried to identify biomarkers for the oocyte microenvironment, to be used as predictive factors of embryo and pregnancy outcomes [17–26]. In a previous study [12], we found that high cfDNA levels in FF samples from individually aspirated follicles at oocyte retrieval day were correlated with poor embryo quality at day 3. Moreover, a recent study reported that elevated plasma cfDNA levels were associated with low chances of pregnancy in women undergoing IVF [27]. However, the potential of FF cfDNA to predict the clinical pregnancy outcome in IVF/intracytoplasmic sperm injection (ICSI) cycles remains to be investigated.

In this study, we quantified cfDNA in FF pools and investigated whether cfDNA levels could be related to women’s ovarian reserve status, COS protocols and ovarian response to stimulation treatment. Then we explored the FF cfDNA potential to predict IVF outcomes such as embryo and clinical pregnancy outcomes. Our results suggest that cfDNA levels in FF are significantly influenced by the ovarian reserve status and the type of gonadotropin treatment. CfDNA quantification in FF pools could provide a new non-invasive and easy method to explore the quality of follicular microenvironment and to predict ovarian response, embryo development and the clinical pregnancy outcome. Therefore, during IVF process, cfDNA could be quantified in FF in order to understand and to improve the personalized patient’s care.

## Materials and Methods

### Patients

This prospective study recruited 100 women enrolled in conventional IVF (n = 31) or ICSI (n = 69) program at the ART-PGD Department of the University Hospital of Montpellier. The patients’ characteristics are detailed in Table 1. The women’s age was 34.3 ± 4.5 years (mean ± SD; range: 23 to 43 years) and the body index mass (BMI) was 23.3 ± 4.2 kg/m^2^ (mean ± SD; range: 17 and 39 kg/m^2^). The infertility length was 3.5 ± 1.7 years (mean ± SD). For 61% of the couples this was the first IVF or ICSI cycle and the remaining 39% of the couples had undergone at least one cycle (mean rank number ± SD: 2.1 ± 1.3). In 11% of the couples, no specific cause of infertility was detected, while in the other couples, male (37%), female (36%) or mixed (16%) factors were identified. Based on the AMH level and AFC at day 3 of menstrual cycle, 94 of the 100 patients had a normal ovarian reserve and 6 had low functional ovarian reserve (LFOR). Basal FSH, LH and E2 levels were quantified also at day 3 of the menstrual cycle in each patient (Table 1).

**Table 1:** Cell-free DNA level in follicular fluid pools according to the patients’ clinical characteristics.

Variable	Mean	n	Min-Max	SD	FF cfDNA (ng/μl)	*p-value*
		(total = 100)			Mean ± SD	
					[95%CI]	
Age (years)	34.3	−	23−43	4.5	−	−
< 37 years	−	64	−	−	1.9 ± 2.7 [0.1–2.5]	0.19 NS
≥ 37 years	−	36	−	−	1.5 ± 1.0 [1.1–1.8]	
BMI (kg/m^2^)	23.3	−	17−39	4.2	−	−
18.5≤BMI<25	−	58	−	−	1.9 ± 2.6 [1.2–2.6]	ref
BMI<18.5	−	10	−	−	1.2 ± 1.1 [0.4–1.9]	0.54 NS
25≤BMI<30	−	24	−	−	1.7 ± 1.6 [1.0–2.4]	0.56 NS
BMI≥30	−	8	−	−	1.6 ± 1.5 [0.3–2.8]	0.93 NS
Infertility length (years)*	3.5	−	1−9	1.7	−	−
1	−	8	−	−	1.1 ± 1.6 [0–2.4]	ref
2−4	−	68	−	−	1.4 ± 1.3 [1.1–1.7]	0.08 NS
≥ 5	−	23	−	−	2.9 ± 3.8 [1.3–4.5]	**0.049**
Infertility aetiology						
Male factor	−	37	−	−	1.5 ± 1.1 [1.1–1.9]	ref
Female factor	−	36	−	−	1.9 ± 2.1 [1.2–2.6]	0.72 NS
Tubal alterations (%)	−	9 (25)	−	−	1.3 ± 1.6 [0.1–2.5]	0.28 NS
Endometriosis (%)	−	21 (58.3)	−	−	2.1 ± 2.5 [0.9–3.2]	0.67 NS
Ovulatory dysfunction (%)	−	1 (2.8)	−	−	−	−
Ovarian disorders (%)	−	4 (11.1)	−	−	2.3 ± 1.3 [0.1–4.4]	0.28 NS
Uterine factor (%)	−	1 (2.8)	−	−	−	−
Mixed infertility	−	16	−	−	1.7 ± 3.1 [0.1–3.4]	0.08 NS
Unexplained infertility	−	11	−	−	2.0 ± 3.7 [0–4.5]	0.23 NS
Primary infertility	−	61	−	−	2.1 ± 2.7 [1.5–2.8]	0.08 NS
Secondary infertility	−	39	−	−	1.1 ± 0.8 [0.8–1.4]	
IVF/ICSI rank number	2.1	−	1−4	1.3	−	−
1	−	39	−	−	1.2 ± 0.9 [0.9–1.5]	0.39 NS
> 1	−	61	−	−	2.1 ± 2.7 [1.4–2.8]	
Baseline evaluation						
FSH (IU/l)*	7.4	−	0.1−19	2.4	−	−
< 10	−	87	−	−	1.6 ± 2.0 [1.2–2.1]	0.42 NS
≥ 10	−	12	−	−	2.4 ± 3.7 [0–4.7]	
LH (IU/l)*	5.7	−	1−11.2	1.9	−	−
3─5	−	32	−	−	1.4 ± 1.3 [0.9–1.8]	ref
< 3	−	5	−	−	2.0 ± 1.0 [0.7–3.3]	0.1 NS
> 5	−	60	−	−	1.8 ± 2.6 [1.1–2.4]	0.7 NS
E2 (pg/ml)*	40.7	−	4−99	17.8	−	−
≤ 45	−	66	−	−	1.8 ± 2.5 [1.2–2.5]	0.56 NS
> 45	−	32	−	−	1.5 ± 1.6 [1.0–2.1]	
AMH (ng/ml)*	2.7	−	0.2−8.6	1.6	−	−
≤ 1	−	5	−	−	4.3 ± 5.0 [0–10.4]	0.06 NS
> 1	−	90	−	−	1.6 ± 2.0 [1.2–2.0]	
AFC*	13.7	−	3−25	5.7	−	−
< 10	−	24	−	−	2.3 ± 2.6 [1.2–3.4]	**0.04**
≥ 10	−	63	−	−	1.5 ± 2.2 [1.0–2.1]	
Normal ovarian reserve	−	94	−	−	1.7 ± 2.3 [1.3–2.2]	ref
Low functional ovarian reserve	−	6	−	−	2.1 ± 1.4 [0.6–3.6]	0.29 NS

FF, follicular fluid

SD, standard deviation

BMI, body mass index

FSH, follicle-stimulating hormone

LH, luteinizing hormone

E2, 17β-estradiol

AMH, anti-Müllerian hormone

AFC, antral follicle count.

*Total number of patients < 100.

P-values: Mann-Whitney test.

In addition, cfDNA was quantified also in FF pools from 17 women with PCOS who were classified according the Rotterdam criteria [28]. The clinical characteristics of PCOS patients are reported separately in S1 Table.

Each patient’s written informed consent for FF sample collection/analysis was obtained on oocyte retrieval day. This study was approved by the Ethical Committee of the Institute for Regenerative Medicine and Biotherapy and the methods were carried out in accordance with the approved guidelines.

### In vitro fertilization protocol and follicular fluid sample collection

Forty-eight patients received a daily GnRH agonist protocol (Decapeptyl, IpsenPharma) and the others an antagonist protocol. These two protocols included ovarian stimulation by recombinant FSH (r-FSH) (Puregon, MSD, Courbevoie, France). The ovarian response to stimulation was monitored by quantifying serum E2 level and by ultrasound assessment of follicular and endometrial growth. The ovarian stimulation length was 10 ± 1.2 day and the total gonadotropin dose was 2414.7 ± 932.5 IU/l (mean ± SD) (Table 2). Ovulation was triggered by a single injection of 250 μg human chorionic gonadotropin (hCG) (Ovitrelle, Merck Serono, Lyon, France), when at least three follicles reached the diameter of 17 mm or more on ultrasound examination.

**Table 2:** Cell-free DNA level in follicular fluid pools according to controlled ovarian stimulation protocols and ovarian response to stimulation.

Variable	Mean	n	Min-Max	SD	FF cfDNA (ng/μl)	*p-value*
		(total = 100)			Mean ± SD	
					[95%CI]	
Agonist protocol**	−	48	−	−	1.4 ± 2.0 [0.9–2.0]	0.09 NS
Antagonist protocol	−	50	−	−	1.8 ± 1.8 [1.3–2.3]	
Ovarian stimulation treatment						
Days of stimulation	10	−	7−14	1.2	−	−
7−10	−	71	−	−	1.5 ± 1.9 [1.0–1.9]	**0.008**
> 10	−	29	−	−	2.4 ± 2.8 [1.4–3.5]	
Total dose of gonadotropins (IU/l)	2414.7	−	875−4950	932.5	−	−
< 3000	−	66	−	−	1.5 ± 2.1 [1.0–2.0]	**0.01**
≥ 3000	−	34	−	−	2.2 ± 2.3 [1.4–3.0]	
Agonist protocol						
Days of stimulation	10	−	8−14	1.1		
8−10	−	37	−	−	1.1 ± 1.1 [0.7–1.4]	0.05 NS
> 10	−	11	−	−	2.7 ± 3.6 [0.3–5.1]	
Total dose of gonadotropins (IU/l)	2324	−	900−4200	797.8		
< 3000	−	34	−	−	1.1 ± 1.1 [0.7–1.5]	**0.049**
≥ 3000	−	14	−	−	2.4 ± 3.2 [0.5–4.2]	
Antagonist protocol						
Days of stimulation	10	−	7−13	1.2		
7−10	−	33	−	−	1.5 ± 1.6 [1.0–2.1]	0.11 NS
> 10	−	17	−	−	2.2 ± 2.3 [1.0–3.4]	
Total dose of gonadotropins (IU/l)	2475.5	−	875–4950	982.7		
< 3000	−	31	−	−	1.6 ± 2.0 [0.9–2.3]	0.13 NS
≥ 3000	−	19	−	−	2.0 ± 1.6 [1.3–2.8]	
Hormonal ovarian response at ovulation triggering						
Peak E2 level (pg/ml)	1793.2	−	341−4768	799	−	−
1000–2000	−	56	−	−	1.8 ± 2.1 [1.2–2.3]	ref
< 1000	−	12	−	−	2.4 ± 3.5 [0.2–4.6]	0.71 NS
> 2000	−	32	−	−	1.4 ± 1.8 [0.8–2.1]	0.23 NS
Progesterone level (ng/ml)	0.8	−	0.1−1.6	0.3	−	−
< 1	−	76	−	−	1.7 ± 2.1 [1.2–2.2]	0.82 NS
≥ 1	−	24	−	−	1.8 ± 2.6 [0.7–2.9]	
LH level (IU/l)	2.0	−	0.1−6.0	1.5	−	−
< 2	−	38	−	−	1.9 ± 2.0 [1.3–2.6]	0.62 NS
≥ 2	−	24	−	−	2.3 ± 3.5 [0.8–3.7]	

FF, follicular fluid

E2, 17β-estradiol

LH, luteinizing hormone

IVF, *in vitro* fertilization

ICSI, intracytoplasmic sperm injection.

**except two mild ovarian stimulations.

P-values: Mann-Whitney test.

Oocyte retrieval was performed by transvaginal ultrasound-guided aspiration 36h after hCG administration and all follicles were aspirated without flushing. All FF samples collected from the same patient were pooled and cumulus-oocyte complexes were isolated for conventional IVF or ICSI procedures.

Before ICSI, cumulus and coronal cells were removed to assess oocyte maturity rate. On average, 9.5 ± 4.7 oocytes (mean ± SD) (S2 Table) were obtained and individually maintained in 30 μl micro-droplets of culture medium (Vitrolife) under oil, at 37°C, in 5% O_2_, 6% CO_2_, 89% N_2_ and in humid atmosphere. Oocytes were considered as normally fertilized if two pronuclei and two polar bodies were observed 18–20 h after microinjection or insemination. Early cleavage was checked at 25 or 27 h after microinjection or insemination, respectively. On day 2 and 3, embryo morphology was evaluated by microscopic observation of morphological criteria, such as number of blastomeres, blastomere regularity and fragmentation rate. Embryo quality was graded from 1 to 4, as described in S3 Table. A top quality embryo (grade 1 and 2) was defined as an embryo with 4–5 or 6–8 regular blastomeres, at day 2 or 3, respectively, and containing less than 20% fragments. At day 3, top quality embryos were selected for transfer or freezing, whereas the others were cultured up to day 5 and frozen by vitrification (Irvine Scientific recommendation), according to their quality, assessed by Gardner scoring [29]. Four weeks after transfer, clinical pregnancy was confirmed by the presence of at least one gestational sac and the visualization of embryonic heart activity on ultrasound examination.

### Follicular fluid preparation

All FF samples from the same patient were pooled and a volume of 15 ml was centrifuged at 3000g for 15 min. Supernatants were filtered with 0.45 μm filters to eliminate cell debris and then stored at -80°C until cfDNA quantification. A total of 117 FF pools were collected for this study.

### Cell-free DNA extraction and quantification by ALU-qPCR

Follicular Fluid pools were prepared for cfDNA quantification as previously reported [30]. Specifically, 20μl of each FF pool was digested with 16 μg proteinase K (PK) (Qiagen) in 20 μl of buffer (25 ml/l Tween 20, 50 mmol/l Tris and 1 mmol/l EDTA) at 50°C for 20 min, followed by PK heat inactivation and insolubilization at 95°C for 5 min. After centrifugation at 10 000g for 5 min, supernatants were removed and stored at -80°C for cfDNA quantification.

The total cfDNA was quantified by qPCR, using ALU 115 primers [30]. Each ALU-qPCR reaction included 1μl of PK-digested FF pool and a reaction mixture containing 0.25 μM of forward and reverse ALU 115 primers and 5 μL of 2X LightCycler480 SYBR Green I master mix (Roche Applied Science, Germany). CfDNA concentration in FF pools was determined using a standard curve obtained by successive dilutions of genomic DNA [30]. A negative control (without template) was integrated in each qPCR plate and each FF pool was analysed in quadruplicate.

To determine which proportion of cfDNA was released from necrotic or apoptotic cells, cfDNA was also quantified by using qPCR with ALU 247 primers. These primers amplify only larger fragments that result from necrosis. This allows the calculation of DNA integrity by using the Q_247_/Q_115_ ratio, which represents the proportion of cfDNA generated by necrosis over total cfDNA. The mean of Q_247_/Q_115_ ratio was 0.14 (SD: 0.16) in follicular fluid samples (n = 117), suggesting that the cfDNA analysed mainly originated from cellular apoptotic events.

### Statistical analysis

Univariate analysis was performed for each variable. Continuous parametric data are presented as mean ± standard deviation (SD) and categorical variables as numbers and percentages. The Mann-Whitney test and Spearman correlations were used to compare cfDNA levels according to quantitative variables, based on the normality of the distribution assessed using the Shapiro-Wilk test. A multivariate analysis was used to model the clinical pregnancy probability. A logistic regression model was fitted in which all variables associated with a p value lower than 0.20 were included in the univariate analysis. Then, a stepwise procedure allowed obtaining the final multivariate model. The ability of FF cfDNA level to predict the clinical pregnancy outcome was determined by constructing the Receiving Operator Curve (ROC) curve and calculating the area under the curve (AUC) with 95% confidence intervals (CI). The sensitivity and specificity for the optimal cut-off were calculated. Statistical tests were performed using the R (version 2.15.2) software. Results were considered significant when p ≤ 0.05.

## Results

### Cell-free DNA level in follicular fluid pools in relation to ovarian reserve status and infertility length

**Fig 1:**
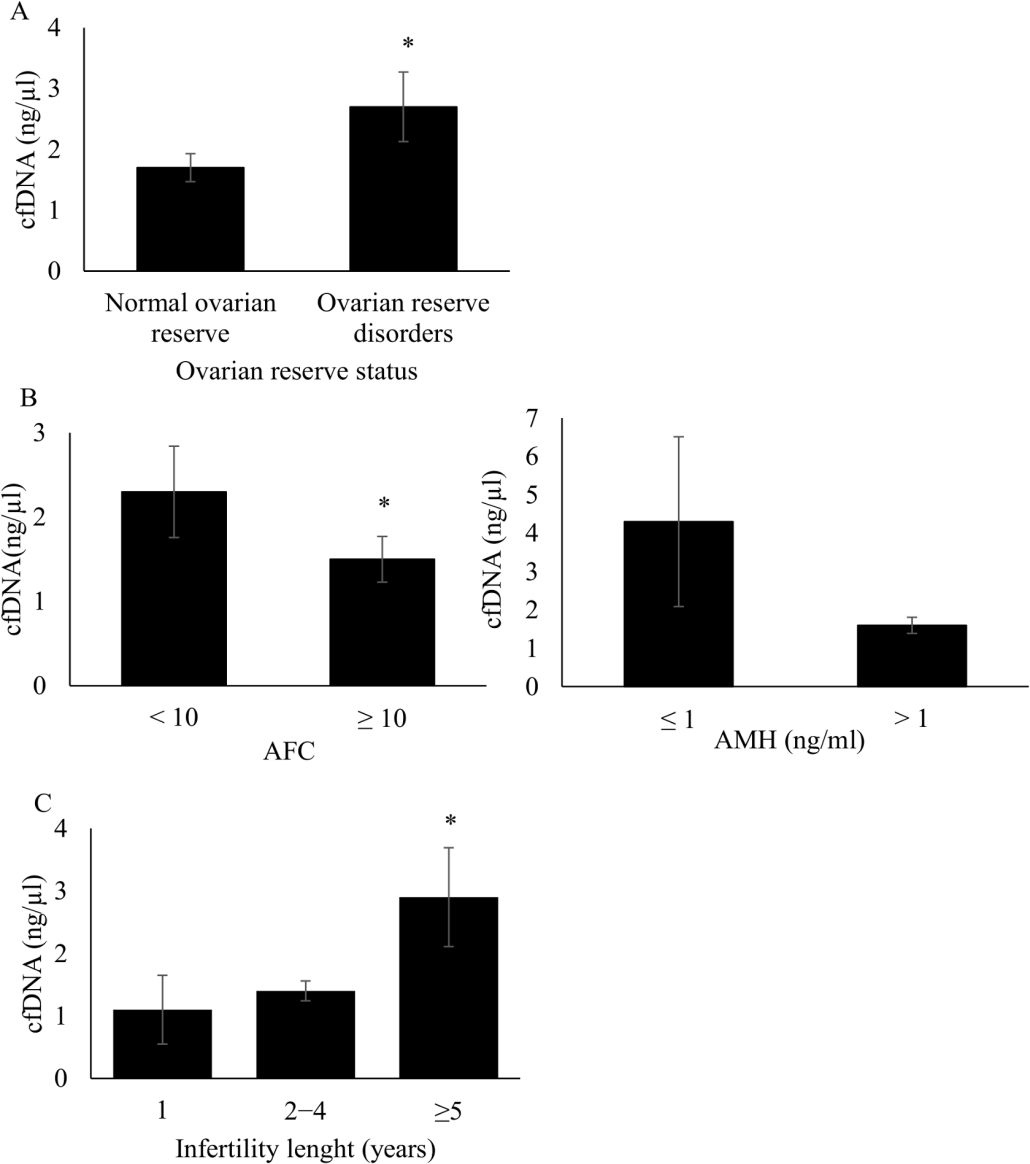
Cell-free DNA level in follicular fluid pools according to the patients’ ovarian reserve status, ovarian reserve parameters and infertility length. **A**, Follicular fluid cfDNA content in patients with normal ovarian reserve versus patients with ovarian reserve disorders (ovarian insufficiency and polycystic ovary syndrome); *p = 0.03. **B**, Follicular fluid cfDNA content according to the ovarian reserve parameters; left panel: AFC (<10 versus ≥ 10, *p = 0.04); right panel: AMH (≤ 1 versus > 1 ng/ml, *p = 0.06). **C**, Follicular fluid cfDNA levels according to the infertility length (1 versus ≥ 5 years, *p = 0.049).

The cfDNA concentration in FF pools of the 17 patients with polycystic ovary syndrome (PCOS) was significantly higher than in FF pools from patients with normal ovarian reserve (n = 94) (2.9 ± 3.1 ng/μl versus 1.7 ± 2.3 ng/μl, p = 0.049) (S1 Fig). Overall, cfDNA levels were significantly higher in FF pools from patients with ovarian reserve disorders (including LFOR and PCOS) than in FF pools from women with normal ovarian reserve (2.7 ± 2.7 ng/μl versus 1.7 ± 2.3 ng/μl, p = 0.03) (Fig 1A).

Given the specific PCOS clinical profile, we decided to exclude these 17 patients from the subsequent analysis. Moreover, cfDNA concentrations were significantly higher in FF pools from patients with low AFC (< 10) than in samples from women with normal AFC (≥ 10) (2.3 ± 2.6 ng/μl versus 1.5 ± 2.2 ng/μl, respectively, p = 0.04) (Fig 1B, left panel and Table 1). Likewise, FF cfDNA level tended to be higher in women with very low AMH serum concentration at day 3 of the menstrual cycle (≤ 1 ng/ml) than in those with AMH > 1 ng/ml (4.3 ± 5.0 ng/μl versus 1.6 ± 2.0 ng/μl, respectively, p = 0.06) (Fig 1B, right panel and Table 1).

Finally, FF cfDNA levels progressively increased with the infertility length and were significantly higher in patients who had been trying to conceive for more than five years compared to women who tried only for one year (2.9 ± 3.8 ng/μl versus 1.1 ± 1.6 ng/μl, p = 0.049) (Fig 1C and Table 1).

### Cell-free DNA concentration in follicular fluid pools according to controlled ovarian stimulation protocol and ovarian response

**Fig 2:**
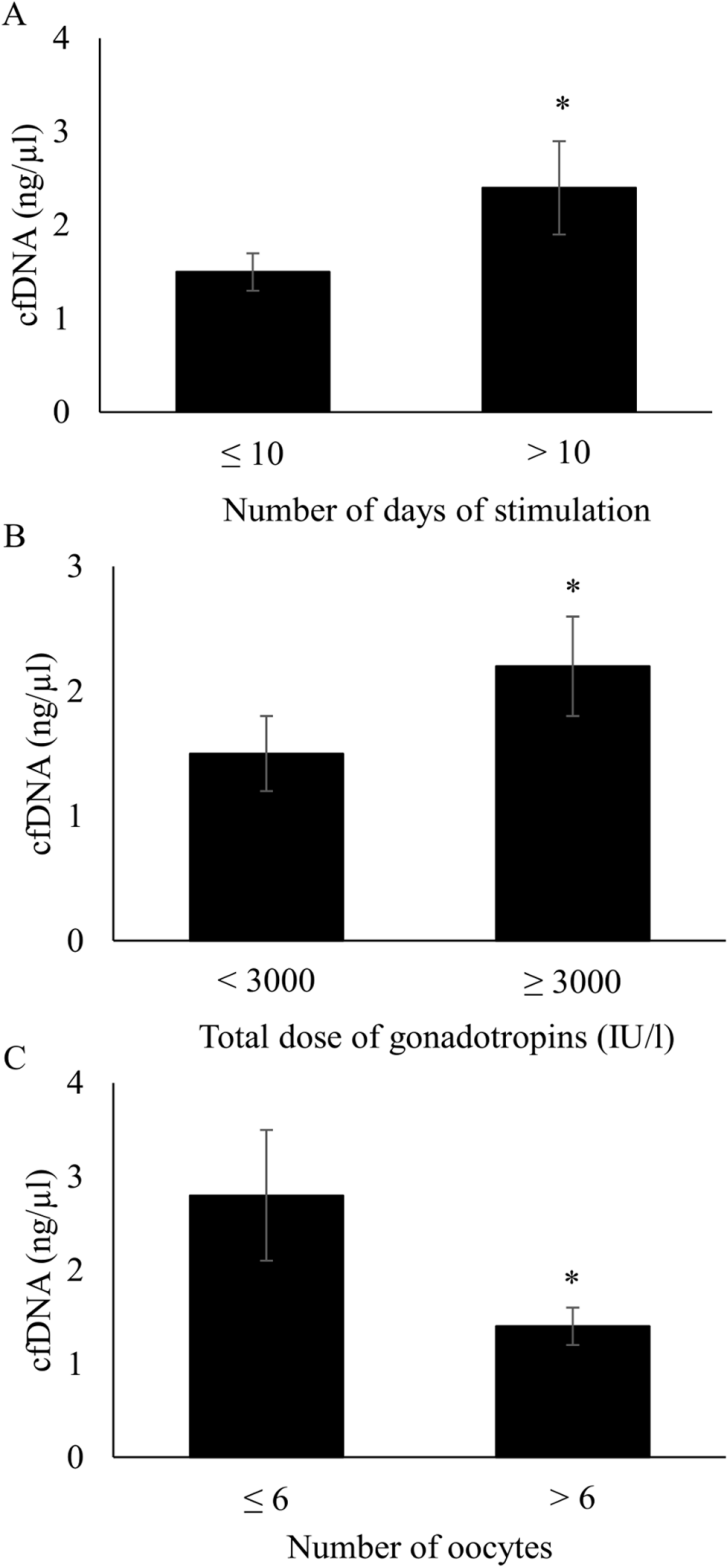
CfDNA level in follicular fluid pools according to the ovarian stimulation protocol and ovarian response. **A**, Follicular fluid cfDNA content according to the length of ovarian stimulation (≤ 10 versus > 10 days, *p = 0.008). **B**, Follicular fluid cfDNA content according to the total dose of gonadotropins (<3000 versus ≥3000 IU/l, *p = 0.01). **C**, Follicular fluid cfDNA content according to the number of retrieved oocytes (≤ 6 versus > 6 oocytes, *p = 0.045).

Follicular Fluid cfDNA level did not vary significantly between women who received GnRH agonists and those treated with antagonists (Table 2). On the other hand, it was significantly higher after long ovarian stimulation (>10 days) than after a short treatment (7–10 days) (2.4 ± 2.8 ng/μl versus 1.5 ± 1.9 ng/μl, p = 0.008) (Fig 2A and Table 2). Likewise, Spearman’s correlation analysis showed that FF cfDNA level was significantly and positively correlated with the ovarian stimulation length (r = 0.2; p = 0.04) (data not shown). Moreover, cfDNA level was significantly higher in FF pools from women who received high total dose of gonadotropins (≥ 3000 IU/l) than in women treated with lower dose (<3000 IU/l) (2.2 ± 2.3 ng/μl versus 1.5 ± 2.1 ng/μl, p = 0.01) (Fig 2B and Table 2). A similar result was obtained when only patients who received an agonist protocol were considered (2.4 ± 3.2 ng/μl versus 1.1 ± 1.1 ng/μl, p = 0.049) (Table 2). In addition, FF pools from patients with a low number of retrieved oocytes (≤6) had a significantly higher cfDNA concentration than those from women with higher number of retrieved oocytes (>6) (2.8 ± 3.5 ng/μl versus 1.4 ± 1.5 ng/μl, p = 0.045) (Fig 2C and S2 Table).

### Cell-free DNA concentration in follicular fluid pools and embryo outcomes

**Fig 3:**
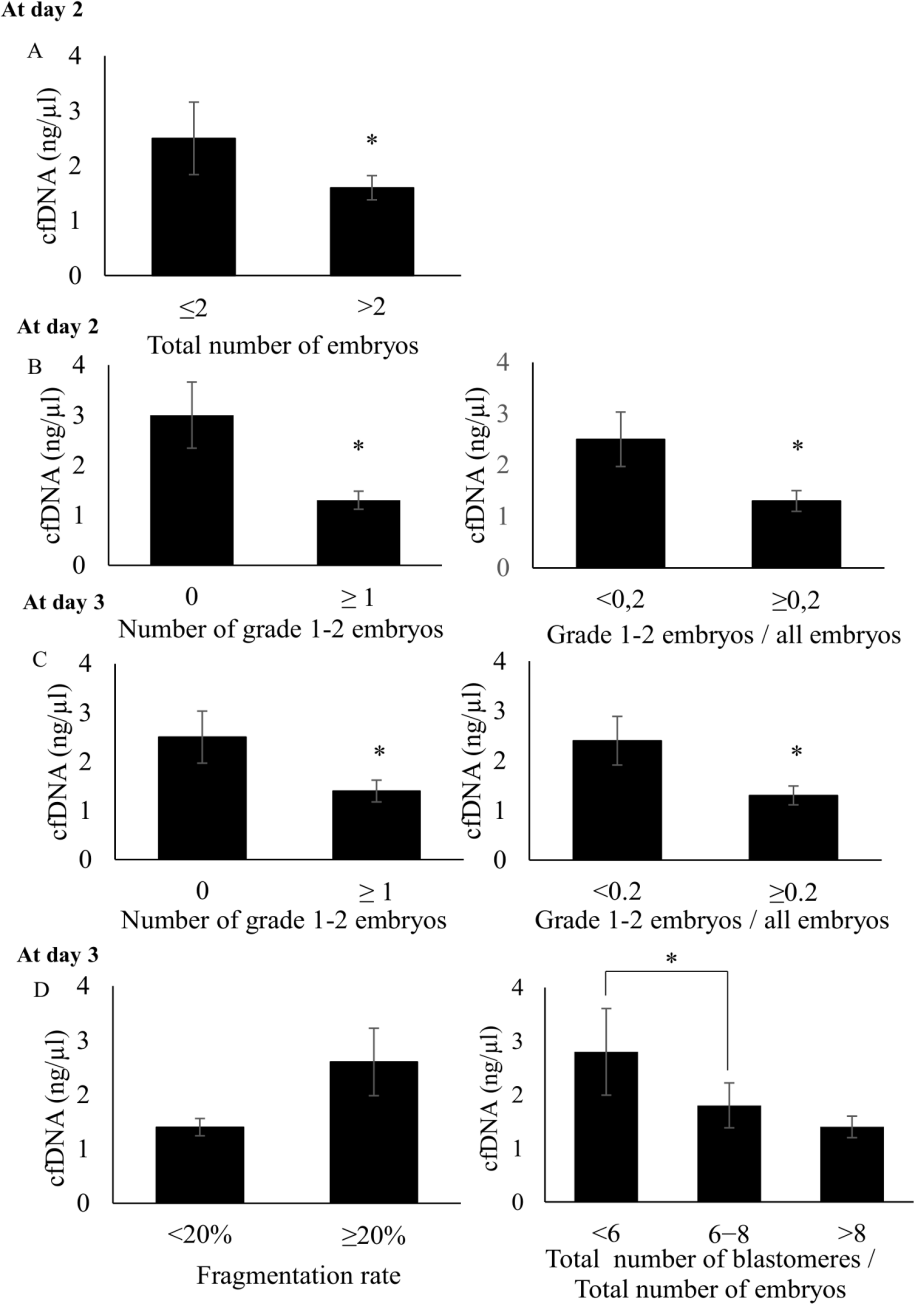
CfDNA level in follicular fluid pools according to the embryo outcome at day 2 and 3. **A**, Follicular Fluid cfDNA content according to the total number of embryos at day 2 (≤ 2 versus > 2, *p = 0.03). **B**, Follicular Fluid cfDNA content according to, left panel: the number of top quality (grade 1–2) embryos per patient (0 versus ≥ 1, *p = 0.002) at day 2, right panel: ratio between number of top quality embryos and total number of embryos (< 0.2 versus ≥ 0.2, *p = 0.04) at day 2. **C**, Follicular Fluid cfDNA content according to, left panel: number of top quality (grade 1–2) embryos per patient (0 versus ≥ 1, *p = 0.006) at day 3, right panel: ratio between number of top quality embryo and total number of embryos (< 0.2 versus ≥ 0.2, *p = 0.02) at day 3. **D**, Follicular Fluid cfDNA content according to, left panel: fragmentation rate at day 3 (<20% versus ≥ 20%, p = 0.18) and right panel: ratio between blastomere number and total embryo number at day 3 (<6 versus 6–8, *p = 0.02).

At day 2 post-fertilization, oocyte cohorts that gave rise to a small number of embryos (≤ 2 embryos) were found to be related to FF pools with significantly higher cfDNA level compared to oocyte cohorts from which at least three embryos were obtained (2.5 ± 2.9 ng/μl versus 1.6 ± 2.0 ng/μl, respectively, p = 0.03) (Fig 3A and Table 3). Moreover, 1.8 ± 1.9 and 1.5 ± 1.5 (mean ± SD) embryos in each embryo cohort (i.e., embryos obtained for each patient) were considered as top quality (grade 1 and 2) at day 2 and day 3, respectively. At these early cleavage stages, cfDNA concentration was significantly higher in FF pools related to embryo cohorts that included only poor quality embryos (grades 3 and 4), compared to those related to cohorts with at least one top quality embryo (at day 2: 3.0 ± 3.4 ng/μl versus 1.3 ± 1.5 ng/μl, p = 0.002; at day 3: 2.5 ± 3.0 ng/μl versus 1.4 ± 1.7 ng/μl, p = 0.006, respectively) (Fig 3B and 3C, left panels and Table 3). Likewise, Spearman’s correlation analysis indicated that there were significant and negative correlations between FF cfDNA concentration and number of top quality embryos (grades 1 and 2) at day 2 and 3 (r = -0.21, p = 0.04; r = -0.21; p = 0.04, respectively) (data not shown). Moreover, cfDNA level was significantly higher in the FF pools related to embryo cohorts with less than 20% top quality embryos at day 2 and 3 compared to those related to embryo cohorts that included more than 20% top quality embryos (day 2: 2.5 ± 3.1 ng/μl versus 1.3 ± 1.5 ng/μl, p = 0.04; day3: 2.4 ± 3.0 ng/μl versus 1.3 ± 1.4 ng/μl, p = 0.02, respectively) (Fig 3B and 3C, right panels and Table 3). In addition, the ratio between number of grade 1–2 embryos and the total number of embryos calculated at day 2 and 3 was significantly and negatively correlated with FF cfDNA level (r = -0.27; p = 0.01 and r = -0.23; p = 0.03, respectively) (data not shown).

**Table 3:** Cell-free DNA levels in follicular fluid pools according to the embryo development outcome at early stages (day 2 and day 3).

Embryo development outcome	Mean	SD	n	FF cfDNA (ng/μl)	*p-value*
				Mean ± SD	
				[95%CI]	
**At day 2**					
Total embryo number	5.3	3.7	─	─	─
≤ 2	─	─	20	2.5 ± 2.9 [1.2–3.9]	**0.03**
> 2	─	─	78	1.6 ± 2.0 [1.1–2.0]	
Grade 1–2 embryos	1.8	1.9	─	─	─
No grade 1−2	─	─	26	3.0 ± 3.4 [1.7–4.4]	**0.002**
≥ 1 grade 1−2	─	─	65	1.3 ± 1.5 [0.9–1.4]	
Grade 1–2 embryos / all embryos	0.3	0.28	─	─	─
ratio < 0.2	─	─	34	2.5 ± 3.1 [1.5–3.6]	**0.04**
ratio ≥ 0.2	─	─	57	1.3 ± 1.5 [0.9–1.7]	
**At day 3**					
Grade 1–2 embryos	1.5	1.5	─	─	─
No grade 1−2	─	─	32	2.5 ± 3.0 [1.4–3.6]	**0.006**
≥ 1 grade 1−2	─	─	59	1.4 ± 1.7 [1.0–1.8]	
Grade 1–2 embryos / all embryos	0.29	0.3	─	─	─
ratio < 0.2	─	─	39	2.4 ± 3.0 [1.4–3.4]	**0.02**
ratio ≥ 0.2	─	─	52	1.3 ± 1.4 [0.9–1.7]	
% fragmentation	0.19	0.11	─	─	─
% fragmentation < 20	─	─	60	1.4 ± 1.3 [1.0–1.7]	0.18 NS
% fragmentation ≥ 20	─	─	31	2.6 ± 3.4 [1.3–3.9]	
Total blastomere number/total embryo number			─	─	─
ratio = 6−8	─	─	45	1.8 ± 2.8 [1.0–2.6]	ref
ratio < 6	─	─	11	2.8 ± 2.7 [1.0–4.6]	**0.02**
ratio > 8	─	─	35	1.4 ± 1.2 [1.0–1.8]	0.39 NS

FF, follicular fluid

SD, standard deviation.

P-values: Mann-Whitney test.

Considering each morphological criterion individually at day 3, cfDNA levels tended to be higher in FF pools related to embryos with high fragmentation rate (≥ 20%) than with low fragmentation rate (<20%) (2.6 ± 3.5 ng/μl versus 1.4 ± 1.3 ng/μl, respectively, p = 0.18) (Fig 3D, left panel and Table 3). Moreover, the ratio between total number of blastomeres and total number of embryos was calculated for each embryo cohort to estimate the global developmental kinetics. At day 3, cfDNA levels were significantly higher in FF pools corresponding to embryo cohorts with a low total blastomere number/total embryo number ratio (<6; delayed development) than in those with normal developmental kinetics (ratio between 6 and 8) (2.8 ± 2.7 ng/μl versus 1.8 ± 2.8 ng/μl, respectively, p = 0.02) (Fig 3D, right panel and Table 3).

### Predictive value of cell-free DNA in follicular fluid pools for clinical pregnancy outcome

**Fig 4:**
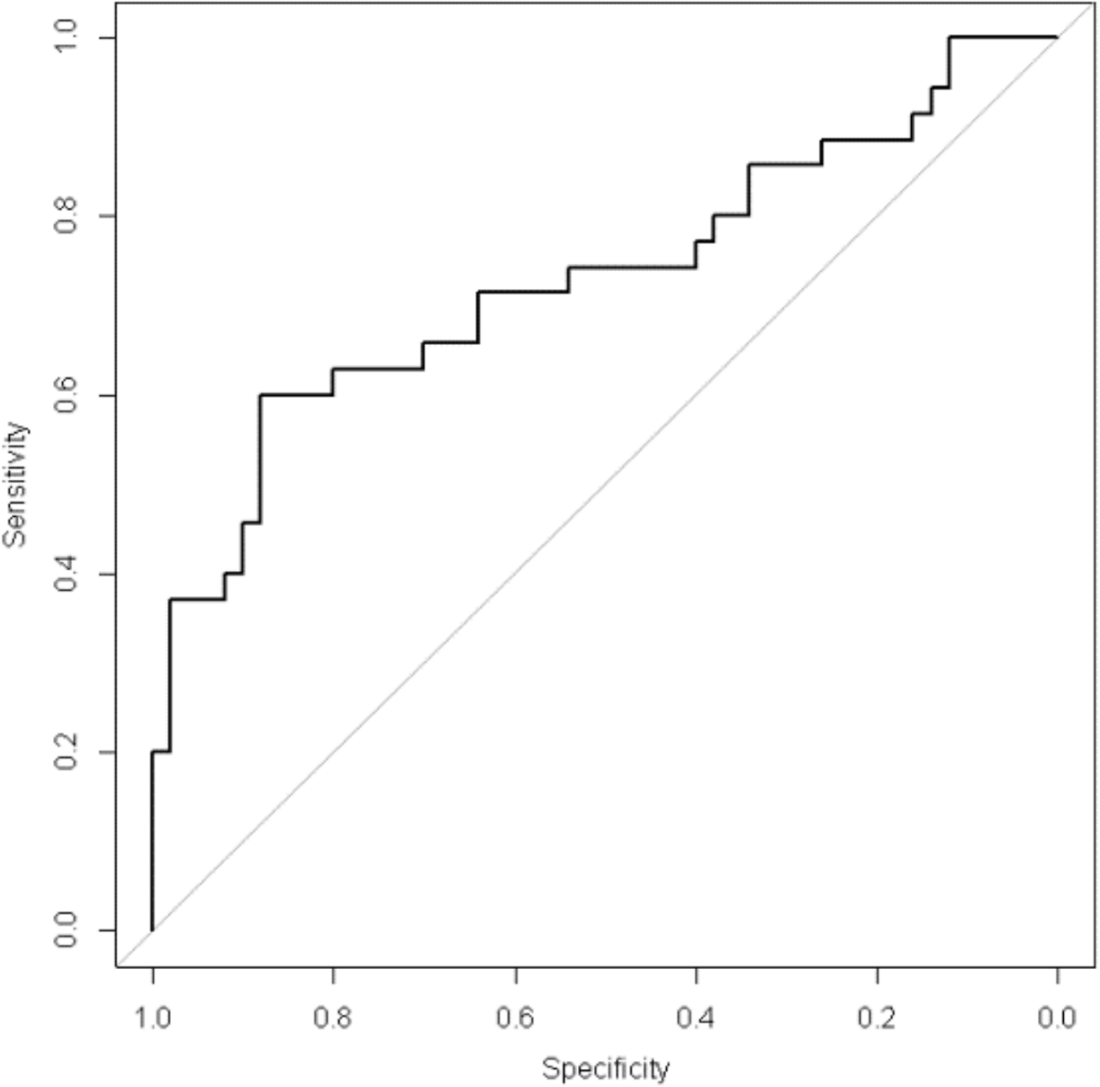
ROC curve to evaluate the predictive value of follicular fluid cfDNA level for clinical pregnancy outcome in a multivariate model (including the rank of IVF/ICSI attempts and the number of embryos): area under the curve = 0.73 [0.66–0.87], sensitivity = 60%, specificity = 88%.

After adjustment for the rank of IVF/ICSI attempts and the number of embryos, FF cfDNA level was significantly and independently associated with the clinical pregnancy outcome [Adjusted Odd Ratio: 0.69 [0.5; 0.96], p = 0.03] (Table 4). The area under the ROC curve, which quantifies the clinical pregnancy prediction potential of FF cfDNA concentration, was 0.73 [0.66–0.87] with 88% specificity and 60% sensitivity (Fig 4). On the other hand, the number of top quality embryos (grades 1 and 2) did not predict significantly the clinical pregnancy outcome (p = 0.42), suggesting that in our population, the predictive value of FF cfDNA level was higher than the number of top quality embryos.

**Table 4:** Multivariate logistic model showing the prediction of clinical pregnancy according to the cell-free DNA level in follicular fluid pools.

Parameters	Crude OR	*p-value*	Adjusted OR	*p-value*
	[95% CI]		[95% CI]*	
**Probability to obtain a clinical pregnancy**				
FF cfDNA (ng/μl)	0.75 [0.55; 1.03]	0.08	0.69 [0.5; 0.96]	**0.03**
IVF/ICSI rank number = 1 vs >1	2.5 [1.0; 6.27]	0.05	3.6 [1.3; 9.8]	0.01
Embryo number	1.15 [1.0; 1.3]	0.04	1.18 [1.01; 1.37]	0.03

OR, odds ratio

*Adjusted to the rank of IVF/ICSI attempts and the number of embryos.

## Discussion

**Fig 5:**
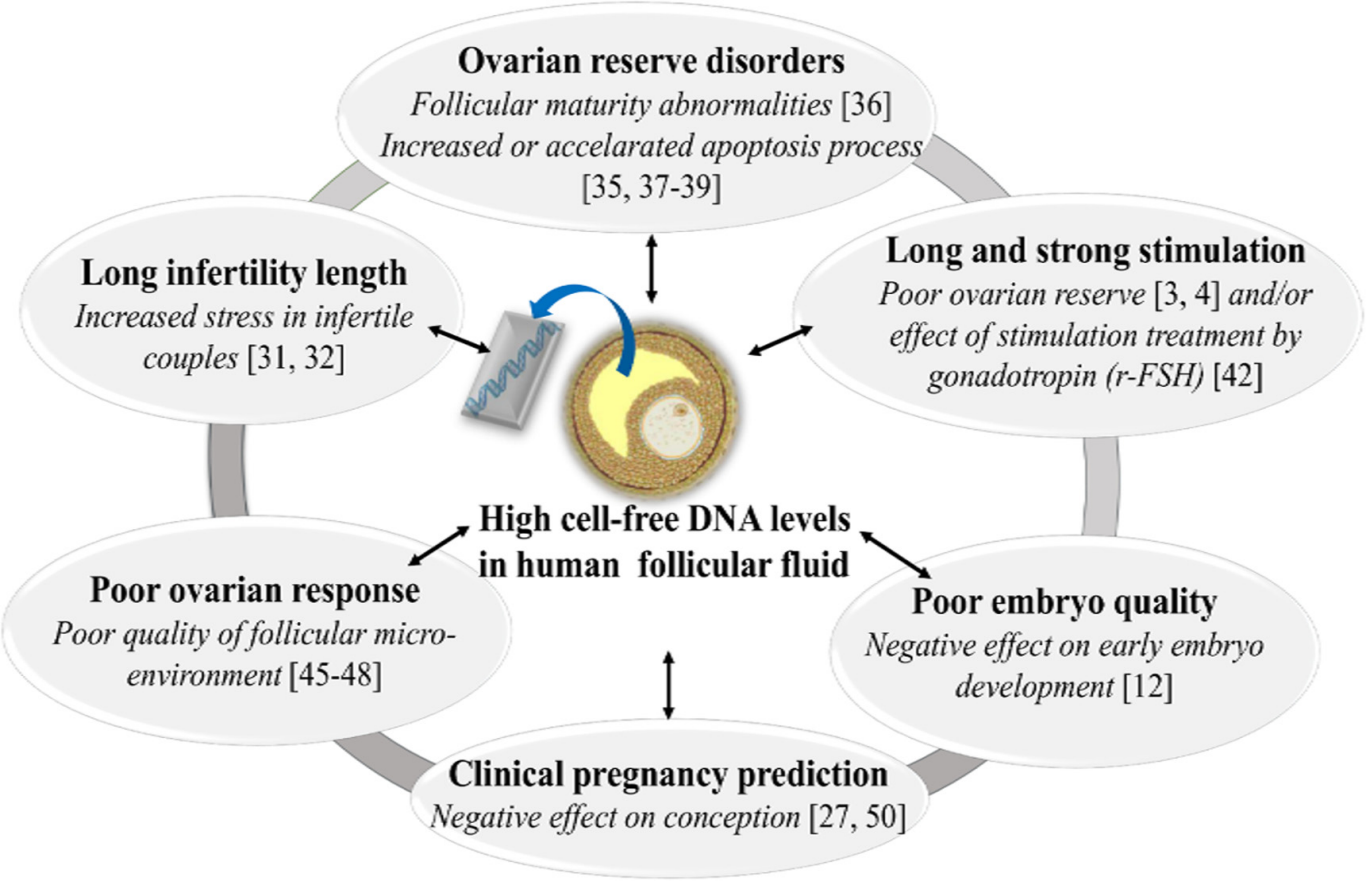
Schematic model summarizing the significant relationships between cell-free DNA levels in human follicular fluid (FF) and: infertility length, ovarian reserve status, ovarian stimulation, ovarian response to stimulation, embryo development and clinical pregnancy outcomes, respectively. High cell-free DNA levels in human FF reflect a poor quality of follicular micro-environment and consequently are related to a poor IVF prognosis. r-FSH, recombinant FSH.

This study demonstrates that cfDNA content in pooled FF samples from the same patient is significantly related to the woman’s ovarian reserve status, suggesting that high FF cfDNA level could reflect a poor follicular micro-environment. It also shows that cfDNA levels were significantly higher in FF pools after a long or strong ovarian stimulation than after a short treatment or stimulation with low doses of gonadotropins. Finally, our data indicate that FF cfDNA could be used to predict the clinical pregnancy outcome (Fig 5). Altogether, our results suggest that FF cfDNA quantification could be considered for improving IVF strategy and outcomes.

CfDNA amount was significantly higher in FF pools from women with long infertility length (more than 5 years). Long infertility length is often associated with increased stress in infertile couples [31, 32] (Fig 5). Interestingly, a recent study reported that blood cfDNA level was higher in patients undergoing IVF and suffering from stress [33]. Therefore, a long period of stress, caused by the absence of pregnancy, could lead to an increase of apoptotic events in follicular cells and ultimately to higher FF cfDNA levels. Moreover, it has been shown that relaxation techniques may be beneficial during IVF process, to reduce plasma cfDNA levels and to improve pregnancy outcomes [33].

CfDNA levels were significantly higher in FF pools from women suffering from PCOS or more generally with ovarian reserve disorders (PCOS and LFOR). PCOS is the most common endocrinopathy in reproductive age women. A variety of biochemical abnormalities have been described in this syndrome, such as hyperinsulinaemia and hyperandrogenism via stimulation of ovarian androgen secretion [34]. Recently, it was reported that high insulin concentration promotes apoptosis in primary cultured rat ovarian granulosa cells [35]. Therefore, high FF cfDNA content in patients with PCOS could be explained by increased apoptosis in granulosa cells due to hyperinsulinaemia. Moreover, we previously reported that cfDNA levels are significantly higher in small follicles compared to large ones [12]. PCOS is associated with follicular maturity abnormalities, such as increased number of small pre-antral follicles [8, 36]. These small follicles could contain high cfDNA levels, thus explaining why cfDNA concentration is high in FF pools from patients with PCOS. We also show that FF cfDNA concentration is high in women with poor ovarian reserve (AFC<10 or AMH≤1 ng/ml) [2, 3]. As ovarian reserve decline is caused by accelerated apoptosis in ovary [37–39], this could lead to an important release of DNA fragments within ovarian follicles. Moreover, in order to optimize their ovarian response, women with poor ovarian reserve receive large gonadotropin doses and at oocyte retrieval day, the practitioner would try to aspirate with more assiduity the smaller follicles to increase number of oocytes. Therefore, in this case follicular fluids from smaller follicles would become proportionally more represented in the pool than in normal responders with a synchronized cohort of larger follicles. These observations suggest that cfDNA content in antral follicles could depend on (i) the basal ovarian status (increased cfDNA in the case of ovarian dysfunction) and/or on (ii) the follicular maturity after recruitment by COS protocols.

Indeed, FF cfDNA level was significantly higher after a long COS protocol (>10 days) or after administration of high doses of gonadotropins (≥ 3000 IU/l). Moreover, the ovarian reserve status strongly influences the ovarian response to COS protocols [8, 40, 41]. For instance, long or strong ovarian stimulation is currently recommended for women at risk of poor ovarian response [3, 4]. Accordingly, patients who received long stimulation or high gonadotropin dose partially overlaps with patients with high intra-follicular cfDNA levels related to low ovarian reserve. Moreover, high FF cfDNA level after long or strong stimulation could represent a true effect of COS protocols, with potential harmful consequences on IVF/ICSI outcomes. For instance, strong supra-physiological gonadotropin doses could induce apoptosis of follicular cells [42], suggesting the necessity to specifically tailor stimulation treatments to each patient’s profile. Conversely, FF cfDNA content did not differ according to the type of COS protocols (agonist versus antagonist). In agreement, similar apoptosis levels were detected in granulosa cells exposed to agonist or antagonist treatments [43].

FF cfDNA concentration was also significantly higher in patients from whom few oocytes were retrieved (≤6) [44] or few embryos obtained (≤2). This observation confirms that high FF cfDNA level is significantly associated with poor ovarian response to COS protocols (Fig 5). Moreover, it suggests that FF cfDNA level is related to both retrieved oocyte quantity and quality, two key features for embryo production. Indeed, it is largely recognized that the follicular environment influences strongly the oocyte developmental competence [45–48]. For this reason, FF cfDNA could represent a new promising biomarker of follicular microenvironment quality. A poor follicular microenvironment, with high cfDNA levels could affect oocyte developmental competence and embryo development, thus leading to IVF failure. As we found that strong or long ovarian stimulation leads to high FF cfDNA level, it could be recommended to adapt the stimulation length and gonadotropin dose to each patient to limit FF cfDNA production. Indeed, the preservation of the follicular microenvironment is primordial to obtain competent oocytes and thus competent embryos.

This study confirms our previous observation [12] that cfDNA levels in FF samples are significantly correlated with embryo quality during early development, when embryos rely on the oocyte maternal reserve (on day 2 and 3). Indeed, cfDNA levels were significantly higher in FF pools related to oocyte cohorts that gave only poor quality embryos, embryos with high fragmentation rate (≥20%) or developmentally delayed embryos (total blastomere number/total embryo number ratio < 6). These poor quality embryos came from oocyte cohorts surrounded by FF containing high cfDNA levels, suggesting a negative effect of a cfDNA-rich follicular environment on embryo quality [12] (Fig 5). In agreement with these results, high mitochondrial DNA level in embryo culture medium was also significantly associated with high fragmentation rate at early embryo cleavages [49].

Finally, FF CfDNA level in a multivariate model predicted significantly the clinical pregnancy outcome with high specificity (88%), independently of the rank of IVF/ICSI attempts and the number of embryos. FF cfDNA level predictive potential was higher than that of the number of top quality embryos (based on morphological criteria). Therefore, this predictive model could be used as a supplemental tool for determining the chance of IVF success. Recently, a significant association between the mitochondrial DNA/genomic DNA ratio in embryo culture medium and implantation outcome was reported [50]. Moreover, Czamanski-Cohen *et al*. [27] found higher cfDNA level in serum samples from patients with low pregnancy rates after IVF. As there is fluid components’ movement between follicles and vasculature [51], DNA fragments could come from massive apoptotic events that occur in the ovaries and that contribute to increasing cfDNA level in FF samples.

In addition, cfDNA quantification in FF pools, fast and easy to perform, could provide an overall picture of the follicular micro-environment quality, influencing IVF outcomes. Therefore, this quantification could be associated with the morphology-based method in order to improve embryo selection for replacement or freezing and consequently the chance of IVF success. This biomarker might constitute a supplemental tool for improving female infertility management and developing a personalized care program.

## Supporting Information

S1 Fig Comparison of cfDNA levels in follicular fluid pools of patients with normal ovarian reserve (n = 94) and patients with polycystic ovary syndrome (PCOS) (n = 17); *p = 0.049. (TIF)

S1 Table Clinical characteristics and ovarian response to stimulation of patients with polycystic ovary syndrome (PCOS) (n = 17). SD, standard deviation; BMI, body mass index; FSH, follicle-stimulating hormone; LH, luteinizing hormone; E2, 17β-estradiol; AMH, anti-Müllerian hormone; AFC, antral follicle count; IVF, *in vitro* fertilization; ICSI, intracytoplasmic sperm injection.
(DOCX)

S2 Table CfDNA level in follicular fluid pools according to oocyte retrieval, fertilization and early cleavage outcomes. SD, standard deviation; MII, oocyte blocked in meiotic metaphase II; GV, germinal vesicle; MI, oocyte blocked in meiotic metaphase I; IVF, *in vitro* fertilization; ICSI, intracytoplasmic sperm injection. P-values: Mann-Whitney test.
(DOCX)

S3 Table Embryo quality classification at day 2 and day 3 post-fertilization. Embryo quality was graded from 1 to 4 (1–2 for top quality embryos; 3–4 for poor quality embryos), based on the following morphological criteria: number of blastomeres, blastomere regularity and fragmentation rate
(DOCX)
