# Supplementary figures and images for: Cell-free DNA in Human Follicular Microenvironment: New Prognostic Biomarker to Predict in vitro Fertilization Outcomes
Source: PLoS One. 2015 Aug 19;10(8):e0136172. doi: 10.1371/journal.pone.0136172 (PMC4545729; doi:10.1371/journal.pone.0136172)

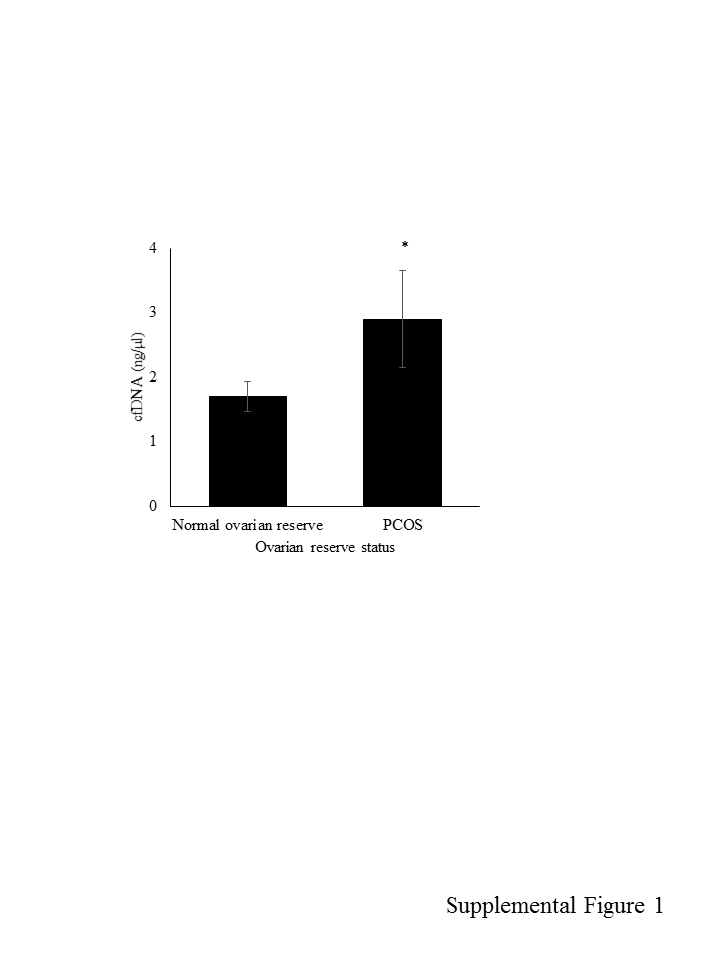

Supplement: S1 Fig — (TIF) [file pone.0136172.s001.tif]
