# Supplementary material for: Cell-free DNA in Human Follicular Microenvironment: New Prognostic Biomarker to Predict in vitro Fertilization Outcomes
Source: PLoS One. 2015 Aug 19;10(8):e0136172. doi: 10.1371/journal.pone.0136172 (PMC4545729; doi:10.1371/journal.pone.0136172)
Supplement: S1 Table — SD, standard deviation; BMI, body mass index; FSH, follicle-stimulating hormone; LH, luteinizing hormone; E2, 17β-estradiol; AMH, anti-Müllerian hormone; AFC, antral follicle count; IVF, in vitro fertilization; ICSI, intracytoplasmic sperm injection. (DOCX) [file pone.0136172.s002.docx]

**S1 Table.** Clinical characteristics and ovarian response to stimulation of patients with polycystic ovary syndrome (PCOS) (n=17).

| Variable | Mean | n | SD |
| --- | --- | --- | --- |
|  |  | (total=17) |  |
| Age (years) | 33.5 | − | 4.2 |
| < 37 years | − | 14 | − |
| ≥ 37 years | − | 3 | − |
| BMI (kg/m^2^) | 26.7 | − | 5.9 |
| 18.5≤BMI<25 | − | 6 | − |
| BMI<18.5 | − | 1 | − |
| 25≤BMI<30 | − | 5 | − |
| BMI≥30 | − | 5 | − |
| Infertility lenght (years) | 3.8 | − | 1.3 |
| 1 | − | 1 | − |
| 2−4 | − | 12 | − |
| ≥ 5 | − | 4 | − |
| Primary infertility | − | 6 | − |
| Secondary infertility | − | 11 | − |
| IVF/ICSI rank number | 1.9 | − | 1.2 |
| 1 | − | 7 | − |
| > 1 | − | 10 | − |
| Baseline evaluation |  |  |  |
| FSH (IU/l) | 5.8 | − | 1.8 |
| LH (IU/l) | 6.9 | − | 3.5 |
| E2 (pg/ml) | 36.3 | − | 15.1 |
| AMH (ng/ml) | 7.4 | − | 3.5 |
| AFC | 26 | − | 11.5 |
| Agonist protocol | − | 13 | − |
| Antagonist protocol | − | 4 | − |
| Days of stimulation | 10 | − | 1.7 |
| Total dose of gonadotropins (IU/l) | 1917.2 |  | 751.7 |
| Hormonal ovarian response at ovulation triggering |  |  |  |
| Peak E2 level (pg/ml) | 2068.9 | − | 847.2 |
| Progesterone level (ng/ml) | 0.7 | − | 0.4 |
| LH level (IU/l) | 2.4 | − | 1.8 |

SD, standard deviation; BMI, body mass index; FSH, follicle-stimulating hormone; LH, luteinizing hormone; E2, 17β-estradiol; AMH, anti-Müllerian hormone; AFC, antral follicle count; IVF, *in vitro* fertilization; ICSI, intracytoplasmic sperm injection.
