# Supplementary material for: Cell-free DNA in Human Follicular Microenvironment: New Prognostic Biomarker to Predict in vitro Fertilization Outcomes
Source: PLoS One. 2015 Aug 19;10(8):e0136172. doi: 10.1371/journal.pone.0136172 (PMC4545729; doi:10.1371/journal.pone.0136172)
Supplement: S2 Table — SD, standard deviation; MII, oocyte blocked in meiotic metaphase II; GV, germinal vesicle; MI, oocyte blocked in meiotic metaphase I; IVF, in vitro fertilization; ICSI, intracytoplasmic sperm injection. P-values: Mann-Whitney test. (DOCX) [file pone.0136172.s003.docx]

**S2 Table.** Cell-free DNA level in follicular fluid pools according to oocyte retrieval, fertilization and early cleavage outcomes.

| Oocyte retrieval, fertilization and early cleavage outcomes | Mean | SD | n | FF cfDNA (ng/µl) | *p-value* |
| --- | --- | --- | --- | --- | --- |
|  |  |  |  | Mean ± SD [95%CI] |  |
| **Oocytes** | 9.5 | 4.7 | − | − | − |
| ≤ 6 | − | − | 25 | 2.8 ± 3.5 [1.4-4.2] | **0.045** |
| > 6 | − | − | 75 | 1.4 ± 1.5 [1.0-1.7] |  |
| **Empty zona pellucida** | 0.4 | 0.8 | − | − | − |
| No Empty zona pellucida | − | − | 87 | 1.5 ± 1.8 [1.1-1.9] | 0.3 NS |
| ≥ 1 Empty zona pellucida | − | − | 24 | 2.4 ± 3.2 [1.2-3.7] |  |
| **Mature oocytes (MII)** | 7.2 | 4.1 | − | − | − |
| < 5 | − | − | 19 | 2.4 ± 3.1 [1.0-3.9] | 0.25 NS |
| ≥ 5 | − | − | 50 | 1.5 ± 1.6 [1.1-2.0] |  |
| Mature oocytes/oocytes | 0.76 | 0.21 | − | − | − |
| ratio < 0.75 | − | − | 26 | 1.5 ± 1.5 [0.8-2.1] | 0.45 NS |
| ratio ≥ 0.75 | − | − | 43 | 2.0 ± 2.4 [1.2-2.7] |  |
| **Immature oocytes (GV, MI)** | 1.8 | 1.9 | − | − | − |
| < 3 | − | − | 67 | 1.9 ± 2.5 [1.3-2.5] | 0.97 NS |
| ≥ 3 | − | − | 33 | 1.4 ± 1.4 [1.0-1.9] |  |
| Immature oocytes/oocytes | 0.18 | 0.18 | − | − | − |
| ratio < 0.25 | − | − | 70 | 1.9 ± 2.5 [1.3-2.5] | 0.26 NS |
| ratio ≥ 0.25 | − | − | 30 | 1.3 ± 1.5 [0.8-1.9] |  |
| **Atretic oocytes** | 0.3 | 0.8 | − | − | − |
| No atretic oocyte | − | − | 85 | 1.7 ± 2.3 [1.2-2.3] | 0.44 NS |
| ≥ 1 atretic oocyte | − | − | 15 | 1.7 ± 1.3 [1.0-2.4] |  |
| Atretic oocytes/oocytes | 0.02 | 0.07 | − | − | − |
| ratio ≤ 0.1 | − | − | 88 | 1.7 ± 2.3 [1.2-2.2] | 0.19 NS |
| ratio > 0.1 | − | − | 12 | 1.9 ± 1.3 [1.0-2.7] |  |
| **Fertilization** |  |  |  |  |  |
| % IVF/ICSI fertilization | 0.65 | 0.3 | − | − | − |
| < 0.65 | − | − | 20 | 1.9 ± 1.7 [1.2-2.7] | 0.27 NS |
| ≥ 0.65 | − | − | 79 | 1.7 ± 2.4 [1.2-2.2] |  |
| **At 25-27 hours after fertilization** |  |  |  |  |  |
| Early cleavage | 1.8 | 2.6 | ─ | ─ | ─ |
| No | ─ | ─ | 36 | 1.9 ± 2.7 [1.0-2.9] | 0.72 NS |
| ≥ 1 | ─ | ─ | 55 | 1.7 ± 2.0 [1.1-2.2] |  |
| Early cleavage/2PN | 0.41 | 0.88 | − | − | − |
| ratio ≤ 0.5 | − | − | 68 | 2.0 ± 2.6 [1.4-2.6] | 0.18 NS |
| ratio > 0.5 | − | − | 21 | 1.0 ± 0.8 [0.7-1.4] |  |
| % fragmentation | 0.07 | 0.09 | ─ | ─ | ─ |
| <10% | ─ | ─ | 31 | 1.3 ± 1.3 [0.9-1.8] | 0.25 NS |
| ≥10% | ─ | ─ | 24 | 2.1 ± 2.6 [1.0-3.2] |  |

FF, follicular fluid; SD, standard deviation; MII, oocyte blocked in meiotic metaphase II; GV, germinal vesicle; MI, oocyte blocked in meiotic metaphase I; IVF, *in vitro* fertilization; ICSI, intracytoplasmic sperm injection. P-values: Mann-Whitney test.
