# Supplementary material for: Cell-free DNA in Human Follicular Microenvironment: New Prognostic Biomarker to Predict in vitro Fertilization Outcomes
Source: PLoS One. 2015 Aug 19;10(8):e0136172. doi: 10.1371/journal.pone.0136172 (PMC4545729; doi:10.1371/journal.pone.0136172)
Supplement: S3 Table — Embryo quality was graded from 1 to 4 (1–2 for top quality embryos; 3–4 for poor quality embryos), based on the following morphological criteria: number of blastomeres, blastomere regularity and fragmentation rate (DOCX) [file pone.0136172.s004.docx]

**S3 Table.** Embryo quality classification at day 2 and day 3 post-fertilization.

| Morphological criteria | grade | | | | |
| --- | --- | --- | --- | --- | --- |
|  | **1** | **2** | **3** | **4** | |
| **Number of blastomeres** |  |  |  |  |  |
| Day 2 | 4−5 | 4−5 | 4−5 | <4 or >5 | − |
| Day 3 | 6−8 | 6−8 | 6−8 | <6 or >8 | − |
| **Blastomere regularity** | Regular | Regular | Regular or | Regular or | Regular or |
|  |  |  | irregular | irregular | irregular |
| **Fragmentation rate (%)** | ≤10 | 10−19 | 20−40 | <40 | >40 |

Embryo quality was graded from 1 to 4 (1-2 for top quality embryos; 3-4 for poor quality embryos), based on the following morphological criteria: number of blastomeres, blastomere regularity and fragmentation rate
